# Persistent Renal Oxidative Stress Despite Mannitol Nephroprotection: The Impact of *Social*‐Single Prolonged Stress in Male and Female Rats Exposed to Cisplatin

**DOI:** 10.1002/cbf.70101

**Published:** 2025-07-08

**Authors:** Juliano Ten Kathen Jung, Isabella Pregardier Klann, Bruna Cruz Weber Fulco, Vanessa Angonesi Zborowski, Gilson Zeni, Cristina Wayne Nogueira

**Affiliations:** ^1^ Laboratory of Synthesis, Reactivity, Pharmacological and Toxicological Evaluation of Organochalcogen Compounds, Department of Biochemistry and Molecular Biology, Center of Natural and Exact Sciences Federal University of Santa Maria Santa Maria RS Brazil

**Keywords:** apostosis, chemotherapy, cisplatin, sex‐differences, stress

## Abstract

Cisplatin (CIS) is a chemotherapeutic agent known for nephrotoxicity through oxidative stress. Cancer treatment is also associated with psychological stress. Repeated exposure to social‐single prolonged stress (social‐SPS) modulates long‐term renal oxidative damage and apoptosis in a sex‐dependent manner in rats treated with cisplatin (CIS), despite mannitol's nephroprotective effects. We investigated whether repeated exposure to social‐single prolonged stress (social‐SPS) modulates long‐term renal oxidative damage and apoptosis in male and female rats treated with CIS and mannitol. Male and female Wistar rats were divided into three groups: control, CIS + mannitol, and CIS + mannitol + social‐SPS. Mannitol was administered 1 h before CIS (2 mg/kg/day, i.p., for 5 days). Social‐SPS was applied at three time points. At postnatal day 68, blood and kidney samples were collected for biochemical and Western blot analyses. Plasma renal biomarkers remained unchanged across groups. However, social‐SPS increased renal lipid peroxidation (TBARS) and protein oxidation (carbonyl content) in both sexes. CIS+social‐SPS decreased catalase activity and altered SOD, GST, and NPSH in a sex‐dependent manner. Only female rats showed increased renal BAX/Bcl2 ratio, indicating apoptosis. In males, Na⁺/K⁺‐K‐ATPase activity correlated positively with NPSH content. Despite mannitol nephroprotection, social stress exacerbated renal oxidative stress. Female rats were more susceptible to apoptosis, suggesting sex‐specific vulnerability to combined CIS and stress exposure. These findings highlight the importance of considering psychological stress and sex as modulators of chemotherapeutic toxicity and may inform future strategies for personalized cancer care.

## Summary

- Cancer treatment extends beyond treatment itself, as psychological stress exposure occurs after treatment outcomes.
- This study investigates *social*‐Single Prolonged Stress (social‐SPS) impacts on kidney oxidative stress in male and female Wistar rats exposed to cisplatin.
- Additionally, it delves into the divergent apoptotic patterns observed in both sexes.
- Our findings indicate that *social*‐SPS exacerbates the toxic side effects of cisplatin, with female rats displaying heightened susceptibility to stress compared to males under this protocol.
- The study reveals no significant changes in hepatorenal biomarkers and oxidative stress in the liver.
- Our study highlights the potential of stress modulation to influence cisplatin toxicity, which may have implications for personalized cancer treatment approaches.

## 1 Introduction

Cisplatin (CIS), a well‐known chemotherapeutic drug, is used in a broad spectrum of cancer treatment [1]. CIS is also known for its pro‐oxidant effects that lead to nephrotoxicity, ototoxicity, and hepatotoxicity [2]. Oxidative stress induced by hepatic and nephrotoxic doses of CIS is characterized by a deregulation of intracellular redox homeostasis and apoptosis [3]. Results regarding sex differences in CIS‐based therapy are controversial; other authors argue negatively [4]. But others found effects of sex on CIS toxicity [5]. Several natural and synthetic antioxidant agents have shown renoprotective properties by modulating oxidative damage and improving biochemical parameters in rat kidney tissue; among these, propolis and its bioactive compounds have been demonstrated to mitigate oxidative injury and hypertension‐related renal damage [6, 7, 8], while synthetic organoselenium compounds and selenium supplementation exhibit significant antioxidative effects in renal and adrenal tissues [9, 10]. Furthermore, innovative approaches, such as stem cell therapy, show promise in attenuating oxidative stress and promoting renal regeneration in diabetic models [11].

Mannitol is a diuretic widely used in chemotherapeutic treatments with CIS to induce forced diuresis as a prophylactic treatment against CIS‐induced nephrotoxicity, even with the underlying mechanisms remaining unclear [12]. Previous reports show animal deaths after CIS administration as an acute outcome, and most of the data available focus on the acute effects of CIS administration in rodents [13]. The use of mannitol to enhance the lifespan of rats and see chronic outcomes has been shown to be a suitable choice.

Cancer treatment is physically and psychologically stressful for people because of chemotherapy and the uncertainty of the disease course [14]. A growing body of evidence indicates the involvement of cancer‐related posttraumatic stress disorder, especially after chemotherapy [15]. Considering CIS renal toxicity in rodent acute protocols and the lack of information about peripheral outcomes after stress exposure, the present study aimed to evaluate whether repeated exposure to social‐single prolonged stress (social‐SPS) modulates renal oxidative stress and apoptosis in male and female rats exposed to cisplatin and treated with mannitol.

## 2 Materials and Methods

### 2.1 Animals

This protocol was conducted using male and female Wistar young rats (aged 21 days) housed in polycarbonate cages, with free access to food (which consisted of commercial feed) (GUABI, RS, BRAZIL) and water. Animals were maintained under controlled room temperature conditions (22 ± 2°C) and a 12‐h light/12‐h dark cycle, with a light cycle turned on at 7:00 a.m. Animals were obtained from the Central Animal Laboratory of the Federal University of Santa Maria (UFSM)—Brazil. The project was approved by the Committee on Care and Use of Experimental Animals Resources of UFSM (#2215180119), ensuring the ethical conduct of the research and the well‐being of the animals, and followed the ARRIVE guidelines.

### 2.2 Drugs

Cisplatin (CIS), *cis*‐diamminedichloridoplatinum II, C‐Platin®; Blau, São Paulo, Brazil, was obtained by a donation from the University Hospital of Santa Maria, Brazil, HUSM. Mannitol as a 20% solution was obtained from Blau Pharmaceutical (Blau, SP, Brazil).

A cocktail of protease inhibitors and the bicinchoninic acid assay (BCA) were purchased from Sigma (Sigma‐Aldrich Company, St. Louis, Missouri, USA). One prestained protein standard was purchased from Bio‐Rad (Bio‐Rad, São Paulo, Brazil). All other chemicals used were of analytical grade or from standard commercial suppliers.

### 2.3 Experimental Protocol

Animals arrived at the Experimental Facility Room on 21 postnatal day (PND) and stayed there for 14 days. At PND 35, the animals (*n* = 48) were randomly divided into three experimental groups of each sex (*n* = 24), as follows: Group I: Control (*n* = 8)—non‐stressed rats received an intraperitoneal (i.p) injection of 0.9% saline solution; Group II (*n* = 8)—non‐stressed rats were exposed to CIS at a dose of 2 mg/Kg (i.p), for 5 consecutive days from PND 35–39, and in the Group III (*n* = 8) rats were exposed to CIS (2 mg/kg at PND 35–39) and subjected to a *social*‐SPS protocol at PND 35, 45, and 55. To increase diuresis and minimize renal toxicity, rats in groups II and III received a 20% mannitol solution at 125 mg/kg i.p. 1 h before CIS administration [16]. No animals subjected to this experimental protocol died.

**Figure 1:**
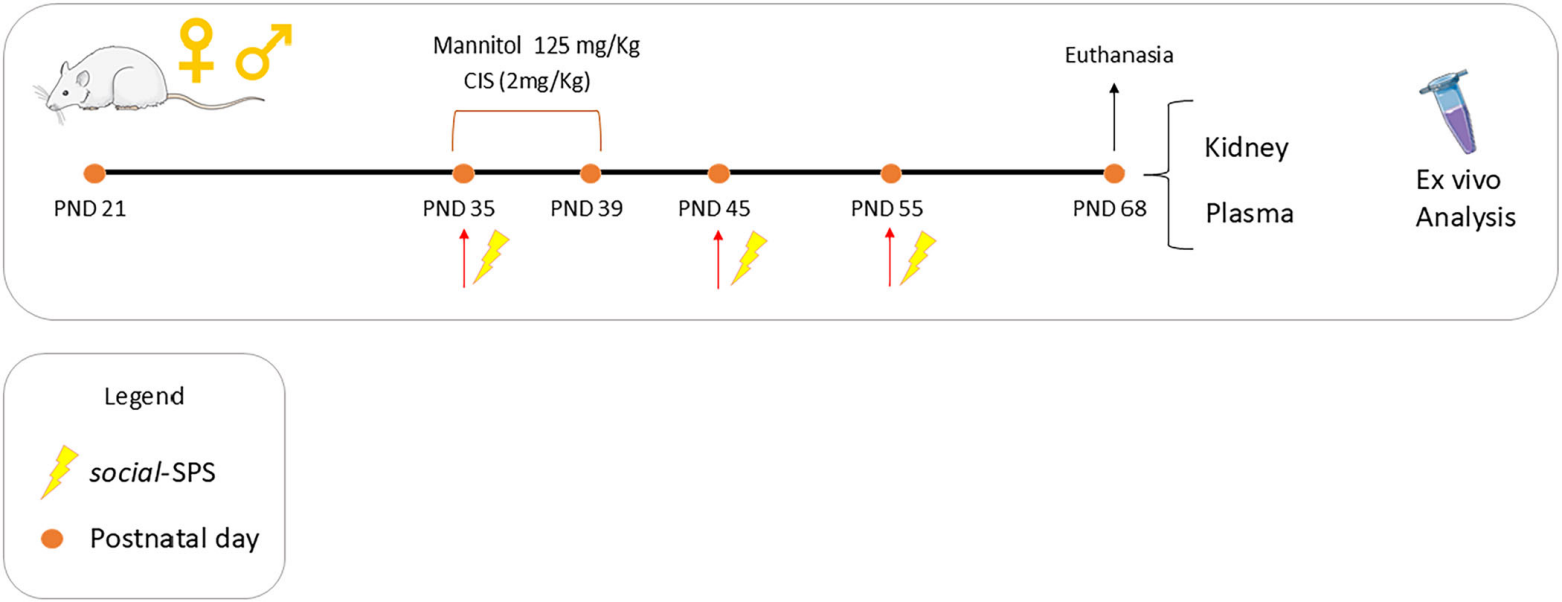
Schematic representation of the experimental protocol. On PNDs 35, male and female Wistar rats were exposed to *social*‐SPS and received a dose of 2 mg/Kg of CIS daily until PND 39. Rats were re‐exposed to *social*‐SPS in PND 45 and PND 55. At PND 68, rats were euthanized, and trunk blood and kidney samples were collected to ex vivo analyses. CIS means cisplatin, PND means postnatal day, *social‐*SPS means social‐single prolonged stress.

At PND 68, animals were killed by decapitation with a commercial guillotine. Kidney and blood samples were collected and kept at −80°C until use. A schematic presentation of the experimental protocol is shown in Figure 1.

#### 2.3.1 Stress Protocol

The *social‐*SPS protocol was designed and modified based on the method described by Liberzon et al [17]. The Liberzon protocol, a robust model designed to induce stress response through three different pathways: psychological (containment), physiological (forced swimming), and pharmacological (ether). In the *social‐*SPS protocol, either exposure was substituted by social instability.

The animals taken from their housing box origin were immobilized individually for 2 h in an acrylic cylinder (6.3 cm height × 6.5 cm width × 23 cm length), with screens at the ends for air entry and exit and holes along the object to facilitate breathing.

Afterward, the animals were immediately placed to swim individually in a cylindrical tank (60 cm height × 20 cm diameter × 25 cm depth) containing clean water at 25°C for 20 min. The room was acclimatized during the execution of the protocol, and the animals were dried with towels and placed in boxes with excess wood shavings, where they remained for 20 min (recovery time), to preserve animal welfare. Then the animals were subjected to the last step of the protocol, which consisted of the relocation of the animal to a new box with unknown rats that also went through the stress protocol. Soon after, the animals remained in the air‐conditioned room until completely dry.

#### 2.3.2 Ex Vivo Assays

##### 2.3.2.1 Renal Function Markers

Plasma uric acid, urea, and creatinine levels were determined as renal function parameters. Blood samples were centrifuged at 2000 × *g* for 10 min to obtain the plasma fraction. These parameters were determined using commercial kits (LabtestDiagnostica, MG, Brazil). The results were expressed as mg/dL.

##### 2.3.2.2 Oxidative Stress Markers

The kidney samples were dissected and homogenized (1:10 w/v) in Tris‐HCl 50 mM at pH 7.4 and centrifuged at 2400 g for 10 min at 4°C to obtain the low‐speed supernatant (S1). S1 freshly prepared was used to determine the levels of reactive species (RS), thiobarbituric acid reactive species (TBARS), and non‐protein thiol (NPSH), and activities of catalase, superoxide dismutase, glutathione S transferase, and Na^+^ K^+^ ATPase. The protein carbonyl content was determined in non‐centrifuged samples.

###### 2.3.2.2.1 Non‐Protein Thiols Content (NPSH)

The levels of the nonenzymatic antioxidant defense NPSH were determined according to the method described by Ellman [18]. An aliquot of S1 was mixed (1:1) with 10% trichloroacetic acid (TCA). After centrifugation (2400 × *g* for 10 min), the protein pellet was discarded, and the free SH‐groups were determined in the clear supernatant. An aliquot of clear supernatant was added in 1 M potassium phosphate buffer, pH 7.4, and 10 mM 5,5′‐dithiobis‐2‐nitrobenzoic acid (DTNB). The color reaction was measured at 412 nm, and NPSH levels were expressed as nmol of NPSH/g tissue.

###### 2.3.2.2.2 Thiobarbituric Acid Reactive Species (TBARS)

Thiobarbituric acid reactive substances (TBARS), a measure of lipid peroxidation, were determined according to Ohkawa et al. [19]. An aliquot of S1 (200 μL) was added to the reaction mixture containing 500 μL of thiobarbituric acid (TBA, 0.8%), 200 μL of sodium dodecyl sulfate (SDS, 8.1%), and 500 μL of acetic acid (pH 3.4) with subsequent incubation at 95 ◦C for 1 h. The reaction product was measured at 532 nm, and the results were expressed as nmol MDA/mg protein.

###### 2.3.2.2.3 Superoxide Dismutase Activity (SOD)

The SOD activity was assayed spectrophotometrically [20]. This method is based on the capacity of SOD in inhibiting the autoxidation of adrenaline to adrenochrome. S1 was diluted 1:10 (v/v) and added to a 0.05 M Na_2_CO_3_ buffer pH 10.2. The enzymatic reaction was started by adding epinephrine. The color reaction was measured at 480 nm. One unit of the enzyme was defined as the amount of enzyme required to inhibit the rate of epinephrine autoxidation by 50% at 26°C. The enzymatic activity was expressed as U/mg protein.

###### 2.3.2.2.4 Catalase (CAT) Activity

The CAT activity was spectrophotometrically assayed by monitoring the H_2_O_2_ consumption at 240 nm [21]. Aliquots of S1 were added to 50 mM potassium phosphate buffer, pH 7.0, and the enzymatic reaction was initiated by adding H_2_O_2_. The enzymatic activity was expressed in Units (1U decomposes 1 μmol H_2_O_2_/min at pH 7 at 25°C)/mg protein.

###### 2.3.2.2.5 Glutathione S Transferase (GST) Activity

The GST activity was measured spectrophotometrically by conjugating GSH to 1‐chloro‐2,4‐dinitrobenzene (CDNB) at 340 nm [22]. An aliquot of 50 μL of S1 was added to 0.1 M potassium phosphate buffer, pH 7.4, 100 mM CDNB, and 100 mM GSH as the substrate. The enzymatic activity was expressed as nmol of conjugated CDNB/min/protein.

###### 2.3.2.2.6 Na^+^, K^+^–Atpase Activity

The Na^+^, K^+^‐ATPase activity reaction mixture contained 6 mM MgCl, 100 mM NaCl, 20 mM KCl, 40 mM Tris–HCl, pH 7.4, and 50 μL of S1 in a final volume of 500 μL. The reaction was initiated by adding ATP to a final concentration of 3.0 mM. Control tubes were carried out under the same conditions with the addition of 1 mM ouabain. The difference between the two assays is calculated as Na^+^, K^+^‐ATPase activity. Released inorganic phosphate (Pi) was measured [23] and expressed as nmol de Pi/mg protein/min.

The Bradford method was used to determine the total protein content in samples [24].

###### 2.3.2.2.7 Protein Carbonyl Content

Carbonyl proteins react with dinitrophenylhydrazine (DNPH) to form dinitrophenylhydrazone [25]. Homogenate was diluted with Tris‐HCl buffer, pH 7.4, in a ratio of 1:10. Aliquots of 1 mL of these dilutions were placed in tubes with 200 μL of 10 mM DNPH in 2 M HCl or only 200 μL of 2 M HCl (blank). Afterward, all tubes were incubated for 1 h at room temperature in the dark and shaken using a vortex mixer every 15 min. After that, 0.5 mL of denaturation buffer (sodium phosphate buffer, pH 6.8, containing 3% SDS), 1.5 mL of ethanol, and 1.5 mL of hexane were added to all tubes. Forthwith, tubes were vortexed for 40 s and centrifuged for 15 min at 2400 × *g*. Supernatants were discarded, and the pellet obtained was separated, washed twice with 1 mL of a mixture of ethanol: ethyl acetate (1: 1, v/v), and dried at room temperature for 2 min. The pellet was immediately dissolved in 1 mL of the denaturing buffer. Absorbance was measured at 370 nm. Results are expressed as nmol carbonyl content/mg protein.

##### 2.3.2.3 Western Blot Analysis

The expression of Bax and Bcl2 in the samples was determined by homogenizing slices in a lysis buffer supplemented with protease and phosphatase inhibitors. The supernatant was separated by differential centrifugation at 10000 xg for 10 min at 4°C, and the S1 fraction enriched in the cytosol was used. Protein concentrations were measured by the BCA kit. Protein (30 µg/µL) was separated on a 12% SDS‐PAGE gel and transferred to a nitrocellulose membrane. Membranes were blocked for 1 and a half hour at room temperature with 5% nonfat milk in Tris‐buffered saline tween‐20 (TBST 0,1%) and after that incubated with primary antibodies for BAX (1:1000, sc‐7480, Santa Cruz Biotechnology, Dallas, TX, USA) and Bcl2 (1:1000, 50E3, Cell Signaling Technology, Danvers, MS, USA) overnight and then washed with TBST 0.1% and incubated for 1 h with correspondent secondary antibody. For protein quantification, bands were scanned and quantified with β‐actin(1:5000, A2228, Sigma‐Aldrich, St. Louis, MO, USA) as an internal control. A chemiluminescence kit (Amersham, São Paulo/Brazil) was used to record the signal (Amersham Imager 600, GE Healthcare Life Sciences). Image J (NIH, Bethesda, MD, USA) software was used to record the optical density of western blot bands.

### 2.4 Statistical Analysis

Data were analyzed by two‐way analysis of variance (ANOVA) [exposure x sex] followed by Tukey's multiple comparison test. When the two‐way ANOVA was not significant, factor sex was disconsidered, and statistical comparisons among the experimental groups were performed using one‐way ANOVA followed by the Tukey test. Results are presented as the mean ± standard error of the mean (SEM). Probability values less than 0.05 (*p* < 0.05) were considered statistically significant. Pearson's correlation coefficient was used to calculate the linear correlation between two variables.

## 3 Results

### 3.1 Neither *Social‐*SPS nor CIS Altered Plasma Biochemical Parameters of Renal Function in Male and Female Rats Treated With Mannitol

Exposure to *social‐*SPS did not alter the levels of urea, creatinine, and uric acid in male and female rats exposed to CIS and treated with mannitol (Table 1). CIS administration in male and female rats treated with mannitol did not alter renal parameters (Table 1).

**Table 1:** Effects of *social‐*SPS on plasma biochemical markers of renal function on male and female rats exposed to CIS and treated with mannitol.

	Control	CIS^a^	CIS + *social‐*SPS	*F*	*p* value	
Male						
Urea	55.880 ± 7.049	62.880 ± 8.116	84.500 ± 18.410	F (2, 21) = 1.470		*p* = 0.253
Creatinine	0.242 ± 0.059	0.225 ± 0.053	0.185 ± 0.048	F (2, 21) = 0.304		*p* = 0.741
Uric acid	2.700 ± 0.470	2.556 ± 0.376	2.763 ± 0.435	F (2, 21) = 0.061		*p* = 0.941
Female						
Urea	41.500 ± 3.185	52.000 ± 2.563	54.000 ± 4.979	F (2, 21) = 3.259		*p* = 0.058
Creatinine	0.100 ± 0.000	0.100 ± 0.000	0.100 ± 0.000	F (2, 21) = 1.000		*p* = 0.384
Uric acid	2.100 ± 0.314	2.175 ± 0.178	2.329 ± 0.340	F (2, 21) = 0.165		*p* = 0.849

*Note:* Values are expressed as the mean ± SEM. Data were analyzed by One‐way ANOVA followed by the Tukey test for post hoc comparison when appropriate.

^a^ CIS exposed rats were treated with mannitol 1 h before exposure.

There was no statistically significant interaction between the effects of exposure and sex on biochemical renal parameters.

### 3.2 *Social‐SPS‐*Induced Renal Oxidative Damage Markers in Male and Female Rats Exposed to Cis and Treated With Mannitol

**Figure 2:**
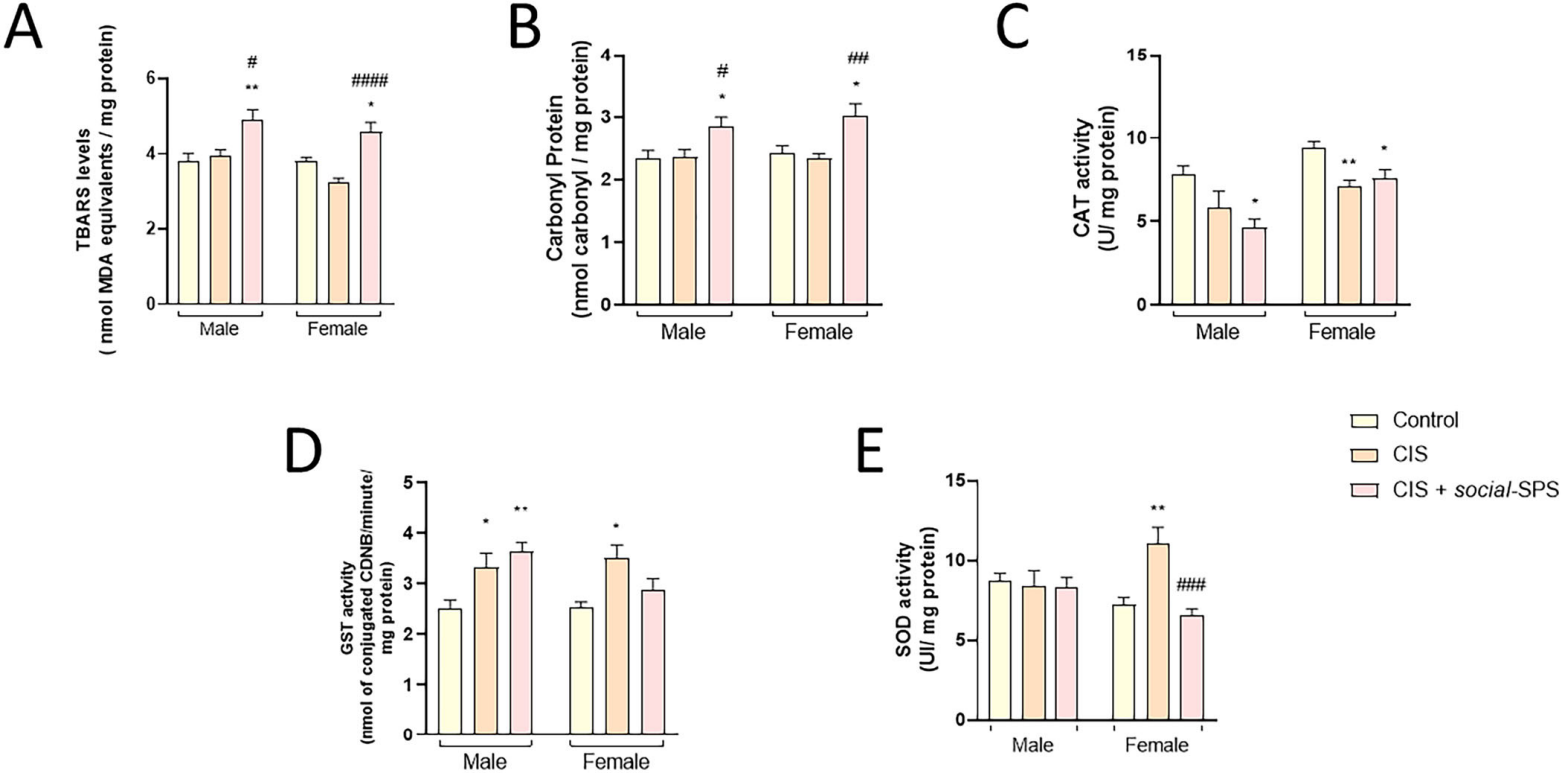
Effects of *social‐*SPS on oxidative stress markers in kidneys of male and female rats exposed to CIS. TBARS levels (A), Carbonyl Protein levels (B), CAT (C), GST (D), and SOD activities (E) in the kidneys of male and female Wistar rats exposed to CIS. Results represent the mean ± S.E.M. of 8 rats per group. Data analysis was carried out through two‐way or one‐way ANOVA followed by Tukey posttest. The symbol denotes a significant difference compared to the control group, *p < 0.05, **p < 0.01. The symbol (#) denotes a significant difference compared to the CIS group, #*p* < 0.05, ##*p* < 0.01, ###*p* < 0.001, ####*p* < 0.0001. CIS means cisplatin, *social‐*SPS means social single prolonged stress.

*Social*‐SPS increased renal TBARS and carbonyl protein levels in male [F_(2,21)_ = 6.525, *p* < 0.0089; F_(2,21)_ = 4.372, *p* = 0.0429] and female rats [F_(2,21)_ = 15.89, *p* = 0.0113; F_(2,21)_ = 6.887, *p* = 0.0201] administered with CIS and mannitol (Figure 2A,B).

CIS administration did not change renal TBARS (Figure 2A) and carbonyl protein levels in male and female rats treated with mannitol (Figure 2B).

There was no statistically significant interaction between the effects of exposure and sex on TBARS and carbonyl protein levels.

### 3.3 *Social‐*SPS Affected the Renal Enzymatic Antioxidant Defenses in Male and Female Rats Exposed to Cis and Treated With Mannitol

Renal CAT activity was decreased in male [F_(2,21)_ = 5.116, *p* = 0.0122] and female rats [F_(2,21)_ = 7.273, *p* = 0.0256] exposed to CIS*+social‐*SPS. CIS exposure decreased renal CAT activity in females [F_(2,21)_ = 7.273, *p* = 0.0043], but this enzyme activity was not altered in male rats treated with mannitol (Figure 2C).

The renal activity of GST was increased in male, but not in female, rats exposed to CIS+*social‐*SPS [F_(2,21)_ = 7.207, *p* = 0.0038]. Male [F_(2,21)_ = 7.207, *p* = 0.0399] and female [F_(2,21)_ = 5.458, *p* < 0.01] rats exposed to CIS and treated with mannitol had an increase in the renal activity of GST (Figure 2D).

There was no statistically significant interaction between the effects of exposure and sex on CAT and GST activities.

### 3.4 Renal SOD Activity Was Affected by *Social‐Sps* and CISis in a Sex‐Dependent Manner

Two‐way ANOVA [F_(2,42)_ = 6.027 *p* = 0.0050] of renal SOD activity showed a statistically significant interaction between factors [exposure x sex].

No statistically significant difference was found for the renal SOD activity in male rats exposed to CIS and treated with mannitol or CIS+*social‐*SPS. Female rats exposed to CIS and treated with mannitol had an increase in SOD activity [*p* = 0.0055] when compared to the respective control group. *Social*‐SPS and CIS‐exposed female rats had the SOD activity decreased when compared to those in the CIS group [*p* = 0.0008] (Figure 2E).

### 3.5 *Social‐*SPS Increased Renal SH Non‐Protein Levels in Male and Female Rats Exposed to Cis and Treated With Mannitol

**Figure 3:**
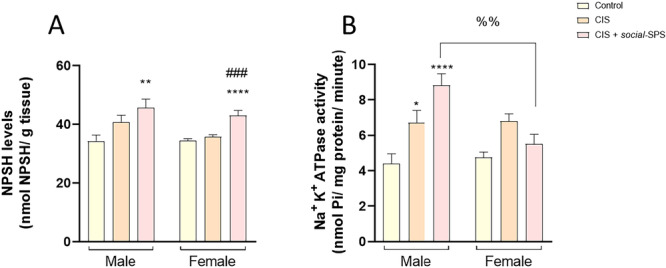
Effects of *social*‐SPS on NPSH content and Na^+^, K^+^‐ ATPase activity in kidneys of male and female rats exposed to CIS. Effects of *social‐*SPS on the NPSH levels (A) and Na^+^, K^+^—ATPase activity (B) in kidneys of male and female Wistar rats exposed to CIS. Results represent the mean ± S.E.M. of 8 rats per group. Data analysis was carried out through two‐way (Na^+^, K^+^—ATPase) or one‐way ANOVA followed by Tukey posttest (NPSH). The symbol (*) denotes a significant difference compared to the control group, *p < 0.05, **p < 0.01, and ****p < 0.0001. The symbol (#) denotes a significant difference compared to the CIS group, ###*p* < 0.001. The symbol (%) denotes a significant difference between male CIS+ *social*‐SPS and female CIS + *social*‐SPS, %% *p* < 0.01. CIS means cisplatin, *social‐*SPS means social single prolonged stress.

Exposure to *social‐*SPS increased the kidney content of NPSH in male [F_(2,21)_ = 5.507, *p* = 0.009] and female rats [F_(2,21)_ = 17.55, *p* = 0.0001] exposed to CIS, but CIS administered to rats did not alter renal NPSH levels in both sexes (Figure 3A).

There was no statistically significant interaction between the effects of exposure and sex on renal NPSH levels.

### 3.6 *Social‐*SPS Abolished the Increase in Renal Na+, K+‐atpase Activity in Females But Not in Male Rats Exposed to Cis and Treated With Mannitol

Two‐way ANOVA [F_(2,42)_ = 7.294 *p* = 0.0019] of renal Na^+^, K^+^‐ATPase activity revealed a statistically significant interaction between factors [exposure x sex]. Renal Na^+^, K^+^‐ATPase activity was increased in male rats exposed to CIS+*social‐*SPS [F_(2,42)_ = 7.294, *p* = 0.0019] when compared to the respective female group.

The groups of male rats exposed to CIS+*Social‐*SPS (*p* = 0.0424) and CIS treated with mannitol (*p* < 0.0001) showed an increase in the renal Na^+^, K^+^‐ATPase activity when compared to the respective control group. *Social‐*SPS, CIS treated with mannitol, or exposure to both did not alter the renal Na^+^, K^+^‐ATPase activity in female rats (Figure 3B) when compared to the respective control group.

Pearson's analyses demonstrated a statistically significant positive correlation between Na^+^, K^+^‐ATPase activity, and NPSH content data for male rats (r = 0.6845, *p* < 0.0001). No significant correlation was found between Na^+^, K^+^‐ATPase activity and NPSH content data for female rats (r = −0.03068, *p* = 0.8792).

### 3.7 *Social‐*SPS and/or CIS Treated With Mannitol Affected Renal Proteins Involved in Apoptosis in a Sex‐Dependent Manner

Two‐way ANOVA [F_(2,24)_ = 7.804, *p* < 0.0001] of renal apoptotic proteins revealed a statistically significant exposure and sex interaction.

**Figure 4:**
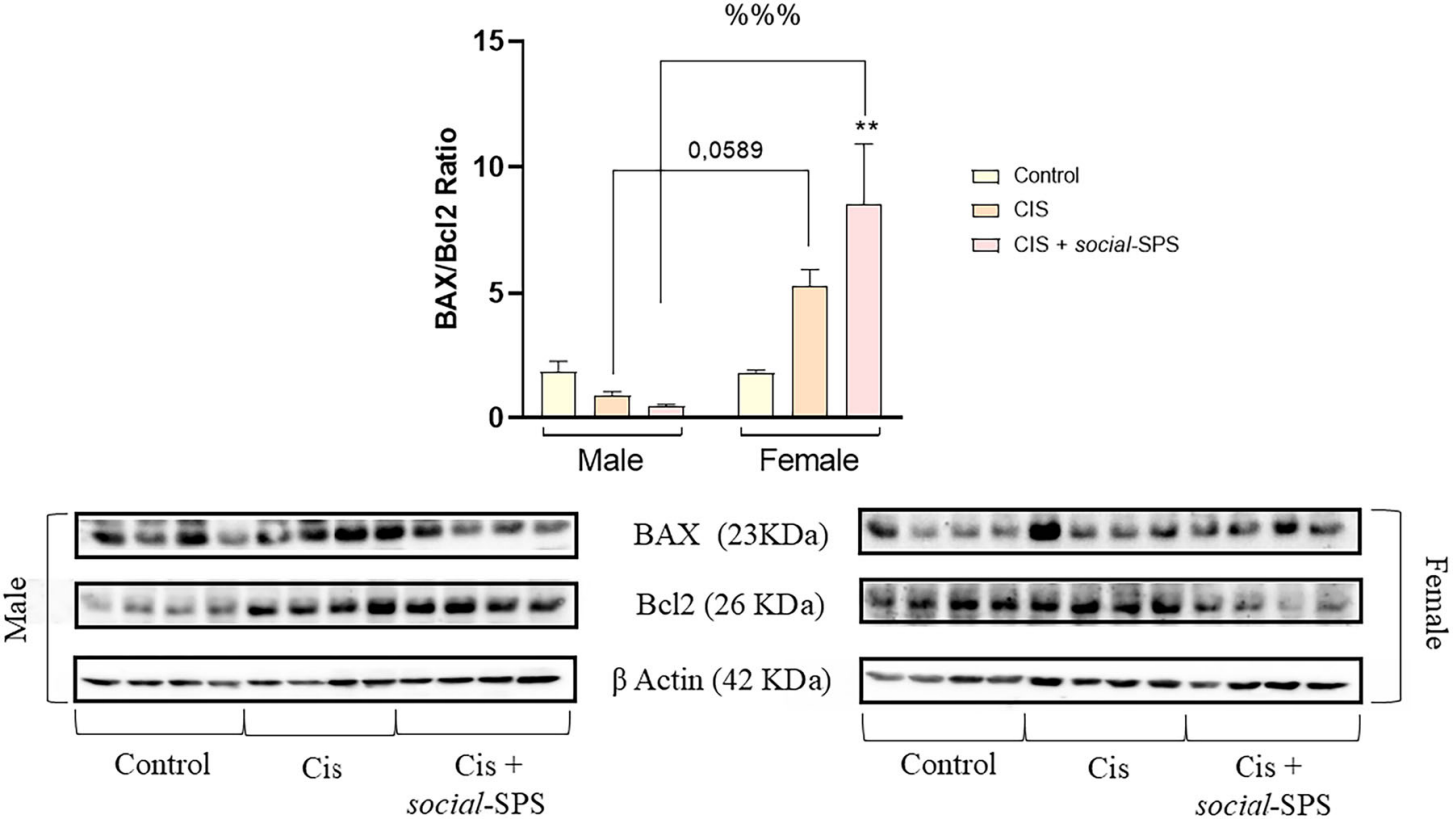
Effects of *social*‐SPS on apoptotic protein levels in male and female rats exposed to CIS. Effects of *social‐*SPS on the BAX/Bcl2 protein level ratio (A) in the kidneys of male and female rats exposed to CIS. Representative bands of BAX/Bcl2 proteins are shown at the bottom of the figure. Results represent the mean ± S.E.M. of 6 rats per group. Data analysis was carried out through two‐way ANOVA followed by Tukey posttest. The symbol (*) denotes a significant difference when compared to the control group, **p < 0.01. The symbol (%) denotes a significant difference when compared to male CIS+ *social*‐SPS and female CIS + *social*‐SPS. %%%*p* < 0.001. CIS means cisplatin, *social‐*SPS means social single prolonged stress.

Figure 4 shows a sex‐dependent pattern of apoptotic profile in the kidney of rats exposed to CIS+*Social*‐SPS.

*Social‐*SPS increased the renal BAX/Bcl2 ratio in female rats exposed to CIS and treated with mannitol when compared to the female control (*p* = 0.0014) and CIS+*social‐*SPS male groups (*p* = 0.0001).

Neither CIS exposure nor CIS+*social‐*SPS increased the renal BAX/Bcl2 ratio in rats when compared to the respective male group.

## 4 Discussion

**Figure 5:**
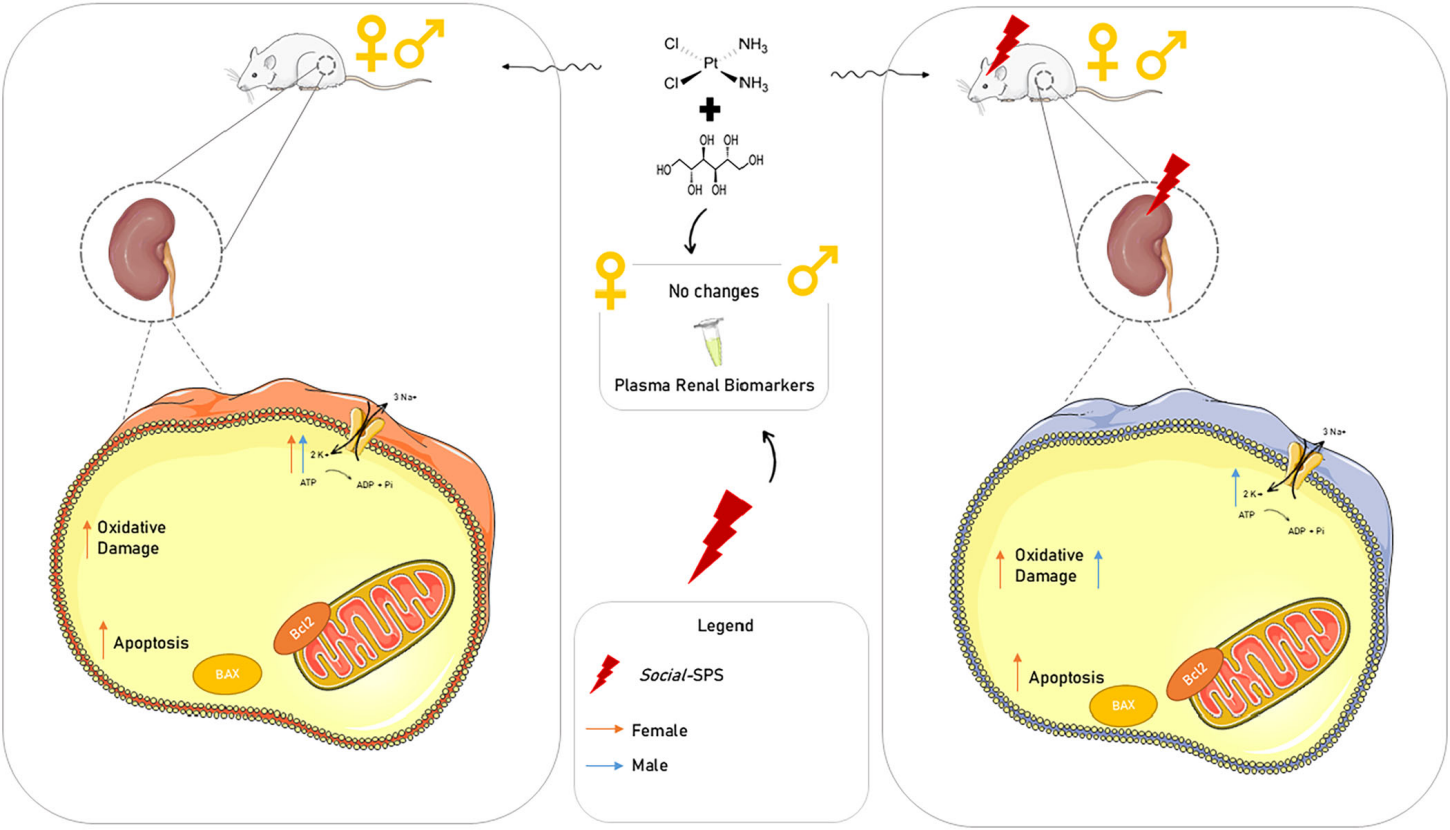
Summary effects of *Social*‐SPS on kidney and plasma of male and female rats exposed to CIS. Effects on male (dark blue arrows) and female (red arrows) rats are represented here. No alterations in renal plasma biomarkers were observed in male and female rats treated with CIS and exposed to social‐SPS. Social‐SPS exposure increased oxidative stress in the kidneys of male and female rats, showing to enhance NPSH content in both sexes and Na^+^, K^+^—ATPase activity only in males. A proapoptotic pattern was showed in kidneys of female rats treated with CIS and exposed to social‐SPS. Adenosine triphosphate (ATP), adenosine diphosphate (ADP), B‐cell lymphoma 2 (Bcl2), Bcl2‐associated X (BAX).

The present results demonstrate that repeated exposures to a *social*‐SPS model induced renal oxidative stress in male and female rats exposed to CIS and treated with mannitol. Regarding sex differences, female rats were more susceptible to renal apoptosis than males when exposed to this protocol (Figure 5). Our results also reveal that despite inducing renal oxidative stress, plasma biomarkers of renal function were not altered in male and female rats exposed to CIS+*social‐*SPS. A plausible explanation for these outcomes is that the acute toxic effects of CIS in plasma biomarkers of male and female rats may not be observable after 29 days of CIS exposure at the dose and administration regimen used in this protocol in animals treated with mannitol. To support this point, evidence has been found to suggest that the onset of acute kidney injury induced by the CIS administration is dependent on time and dose [26], a single high dose or repeated low doses [27]. Accordingly, our previously published studies have demonstrated the increase in plasma biomarkers when rats were exposed to a single administration of CIS at a higher dose (6 mg/kg) than the one used in this protocol (2 mg/kg, 5 days) [28]. Notably, John et al [29] treated young rats with the same dose of CIS (2 mg/Kg for 5 days) and administered mannitol (125 mg/Kg) 1 h before CIS to minimize renal toxicity, reporting no loss of animals.

It is known that oxidative stress plays a critical role in the pathogenesis of kidney injury [30]. Our results show that CIS did not alter TBARS and carbonyl protein levels in the kidneys of male and female rats treated with mannitol in a long‐term way but associated with *social‐*SPS exposure increased oxidative markers in this tissue, demonstrating that stress induced renal redox alterations. Benchimol de Souza et al [31] observed a decrease in the number of nephrons in male Wistar rats subjected to a repeated immobilization stress protocol, without a concurrent increase in serum creatinine levels. Moreover, Miller [32] demonstrated the negative impact of H_2_O_2_ in both renal development and function, emphasizing its role in reducing nephron number. In short‐term protocols, CIS typically inhibits renal CAT activity in an experimental protocol in which mannitol was not administered to rats [33]. Our findings are consistent with existing knowledge, particularly in female rats, twenty‐9 days after exposure to CIS. Although data on sex differences in renal SOD activity between male and female rats, one study reported [34] that extracellular SOD plays a critical role in preserving renal function in a model of renal hypertension in female rats. This underscores the importance of considering sex‐specific responses in understanding the impact of oxidative stress on renal health.

An increase in the non‐protein antioxidant system suggests the active involvement of the kidney in detoxification processes [35]. Notably, both male and female rats in *social*‐SPS + CIS groups exhibited increased NPSH content. While exposure to SPS is recognized for decreasing the GSH/GSSG ratio in central areas in rodents [36], its peripheral effects remain unclear. The enzyme GST, a key player in detoxification, demonstrated increased activity in the CIS group for both sexes and in the male CIS+*social‐SPS* group. CIS acute protocols have been reported to reduce GST activity when mannitol or another diuretic is not used to mitigate CIS‐induced renal toxicity [37]. However, considering the twenty‐9 days post the last administration of CIS and the use of mannitol, we propose that the enzyme recovers from its initial reduced activity, contributing to the continued detoxification of residual CIS. The sulphydryl transporter Na^+^, K^+^‐ATPase activity is correlated to increased sodium transport, a phenomenon common in nephrotic animals [38]. Moreover, Na^+^, K^+^‐ATPase activity depends on the cellular redox status [39]. Interestingly, the activity of Na^+^ and K^+^‐ATPase was increased in CIS treated with mannitol and CIS+*social‐SPS*, but in the kidney of exclusively male rats, indicating a sex‐specific pattern. Furthermore, among male rats, a positive correlation was observed between NPSH content and Na^+^, K^+^‐ATPase activity.

CIS is widely recognized for its proapoptotic effects [27]. Our results reveal a notable sex disparity in apoptosis, particularly with CIS increasing the BAX/Bcl2 ratio in the kidney of female rats, regardless of exposure to *Social‐SPS*. Previous research by Herrera Gutiérrez et al. [40] indicates that female rats manifest a higher expression of genes associated with the initiation of apoptosis, starting from sexual differentiation and hormonal changes, in comparison to their male counterparts [41]. Notably, considering the potential anti‐inflammatory role of apoptosis, it is plausible that male rats could be more susceptible to an inflammatory pattern [42], offering a potential explanation for our observed apoptotic pattern results. Interestingly, CIS administration showed more pronounced redox effects on the kidneys of females, with minimal impact on males, demonstrating a sex‐dependent response pattern.

Stress protocols have predominantly focused on exploring alteration within the central nervous system (CNS), exemplified by models such as the single prolonged stress (SPS) or chronic unpredictable mild stress (CUMS), which aim to mimic conditions such as posttraumatic stress disorder, depression, and anxiety. Whereas stress exposure has been extensively studied for its impact on redox alterations in the brain, such as elevated TBARS levels and decreased GSH/GSSG ratio in the hippocampus of male rats [43]. The discussion of redox changes in peripheral organs like the kidney has been comparatively limited in stress exposure protocols. A protocol using chronic cold exposure in male Wistar rats demonstrated an increase in MDA content and GST activity in the kidney, along with various changes in enzymatic parameters, indicating a redox imbalance in this tissue [44]. Furthermore, investigations reveal that exposure to restraint stress in both rats and mice can lead to oxidative damage in the peripheral blood [45]. Despite these findings, the emphasis in redox alterations in peripheral organs in the context of stress exposure remains a less‐explored area in current research protocols.

To the best of our knowledge, this is one of the few studies examining the impact of exposure to *social‐*SPS pre‐exposed to CIS in male and female rats treated with mannitol, with the assessment conducted twenty‐9 days post CIS exposure. The results collectively indicate that CIS/mannitol did not cause oxidative stress in the kidneys of male rats. In contrast, female rats exhibited susceptibility to CIS despite treatment with mannitol, and repeated exposure to the *Social‐SPS* paradigm exacerbated oxidative stress in the kidneys of male and female rats. Notably, the temporal aspect emerged as a crucial factor in CIS outcomes, as demonstrated by the absence of alterations in renal function markers in plasma. Also, a limitation of our study is the absence of histological (H&E) analysis, which could have complemented the biochemical and molecular findings with structural evidence of renal damage. Acknowledging the limitations of these findings, future studies are necessary to elucidate the specific involvement of CIS/mannitol and its outcomes in a long‐term context, as well as its interaction with stress protocols in peripheral organs.

In conclusion, the present study demonstrates that repeated exposures to a *social*‐SPS induced renal oxidative stress in male and female rats exposed to CIS and treated with mannitol. These findings reinforce the importance of psychological stress management during chemotherapy and highlight the need for sex‐specific approaches to minimize treatment‐related toxicity. While animal studies are not directly translational to human outcomes, the results support the importance of managing stress during cancer treatment and suggest that sex‐specific strategies may improve patient outcomes.

## Author Contributions

Juliano Ten Kathen Jung, Bruna Cruz Weber Fulco, and Cristina Wayne Nogueira designed the present study. Juliano Ten Kathen Jung, Isabella Pregardier Klann, Bruna Cruz Weber Fulco and Vanessa Angonesi Zborowski carried out molecular analyses. J.T.K.J. wrote the original draft, Gilson Zeni and Cristina Wayne Nogueira revised the manuscript critically. All authors approved the final version of the manuscript.

## Ethics Statement

This study was performed and approved by the Committee on Care and Use of Experimental Animals Resources of UFSM (#2215180119), ensuring the ethical conduct of the research and the well‐being of the animals.

## Conflicts of Interest

The authors declare no conflicts of interests.

## Data Availability

The data that support the findings of this study are available from the corresponding author upon reasonable request.
